# Anti-Müllerian Hormone and Testicular Function in Prepubertal Boys With Cryptorchidism

**DOI:** 10.3389/fendo.2018.00182

**Published:** 2018-04-25

**Authors:** Romina P. Grinspon, Silvia Gottlieb, Patricia Bedecarrás, Rodolfo A. Rey

**Affiliations:** ^1^Centro de Investigaciones Endocrinológicas “Dr. César Bergadá” (CEDIE), CONICET – FEI – División de Endocrinología, Hospital de Niños Ricardo Gutiérrez, Buenos Aires, Argentina; ^2^Departamento de Histología, Biología Celular, Embriología y Genética, Facultad de Medicina, Universidad de Buenos Aires, Buenos Aires, Argentina

**Keywords:** anti-Müllerian hormone, gonadotropins, hypogonadism, Sertoli cells, testosterone, undescended testes

## Abstract

**Introduction:**

The functional capacity of the testes in prepubertal boys with cryptorchidism before treatment has received very little attention. The assessment of testicular function at diagnosis could be helpful in the understanding of the pathophysiology of cryptorchidism and in the evaluation of the effect of treatment. Anti-Müllerian hormone is a well-accepted Sertoli cell biomarker to evaluate testicular function during childhood without the need for stimulation tests.

**Objective:**

The aim of the study was to assess testicular function in prepubertal children with cryptorchidism before orchiopexy, by determining serum anti-Müllerian hormone (AMH). We also evaluated serum gonadotropins and testosterone and looked for associations between testicular function and the clinical characteristics of cryptorchidism.

**Materials and methods:**

We performed a retrospective, cross-sectional, analytical study at a tertiary pediatric public hospital. All clinical charts of patients admitted at the outpatient clinic, and recorded in our database with the diagnosis of cryptorchidism, were eligible. The main outcome measure of the study was the serum concentration of AMH. Secondary outcome measures were serum LH, FSH, and testosterone. For comparison, serum hormone levels from a normal population of 179 apparently normal prepubertal boys were used.

**Results:**

Out of 1,557 patients eligible in our database, 186 with bilateral and 124 with unilateral cryptorchidism were selected using a randomization software. Median AMH standard deviation score was below 0 in both the bilaterally and the unilaterally cryptorchid groups, indicating that testicular function was overall decreased in patients with cryptorchidism. Serum AMH was significantly lower in boys with bilateral cryptorchidism as compared with controls and unilaterally cryptorchid patients between 6 months and 1.9 years and between 2 and 8.9 years of age. Serum AMH below the normal range reflected testicular dysfunction in 9.5–36.5% of patients according to the age group in bilaterally cryptorchid boys and 6.3–16.7% in unilaterally cryptorchid boys. FSH was elevated in 8.1% and LH in 9.1% of boys with bilateral cryptorchidism, most of whom were anorchid. In patients with present testes, gonadotropins were only mildly elevated in less than 5% of the cases. Basal testosterone was mildly decreased in patients younger than 6 months old, and uninformative during childhood.

**Conclusion:**

Prepubertal boys with cryptorchidism, especially those with bilaterally undescended gonads, have decreased AMH production. Although serum AMH may fall within the normal range, there is a considerable prevalence of testicular dysfunction during childhood in this frequent condition.

## Introduction

Cryptorchidism is one of the most frequent congenital malformations in the male, with a prevalence at birth ranging from 1.6 to 5.7% (1–4), and up to 9% in Denmark (5). In patients with a history of cryptorchidism, the risk for infertility (6–8) and testicular cancer (7, 9) is highly increased.

Cryptorchidism is the consequence of the lack or insufficiency of the process of testicular descent taking place during fetal life. The testes are initially formed near the kidneys and descend following a two-phase process (10); in the first phase, between the 8th and 15th fetal weeks, the testes are anchored to the internal inguinal ring by the gubernaculum. A Leydig cell factor named insulin-like 3 (INSL3) and its receptor RXFP2 are major regulators of gubernacular development (11). In addition, the androgen-regulated regression of the testicular cranial suspensory ligament also seems to play a role (12). Anti-Müllerian hormone (AMH) has also been suggested to participate, although this remains controversial (13). In the second phase, the testes migrate from the internal inguinal ring to the scrotum, mainly driven by the effect of androgens and intra-abdominal pressure (14). This phase is usually completed in humans by the time of birth; therefore, prematurity is associated with a higher incidence of cryptorchidism (5). Spontaneous descent may still occur in the first months of postnatal life (4, 5), and re-ascent can occur later in life probably associated with the development of the cremasteric reflex and the existence of a remnant of the processus vaginalis preventing normal elongation of the spermatic cord, leading to the concept of acquired cryptorchidism (4, 15).

While a large bibliography exists on the controversies regarding the most adequate treatment for cryptorchidism and its timeliness (16, 17), it is surprising that less attention has been given to the functional capacity of the prepubertal testes before treatment. It is clear that cryptorchidism may be a sign of several conditions with different underlying pathogeneses (7). In the vast majority of the cases, the sign is treated—i.e., the abnormal position is repaired—without knowing the degree of affectation of the gonadal axis. Assessing testicular function at the time of diagnosis may help in the understanding of the pathophysiology of cryptorchidism in each patient and in the ensuing evaluation of the effect of treatment.

Because the hypothalamic–pituitary–gonadal axis undergoes a relative quiescence after the age of 6 months and during childhood (18, 19), classical markers like testosterone and gonadotropins are of little use in the assessment of testicular function in the prepubertal boy. Conversely, Sertoli cells maintain an active secretory activity and serum levels of Sertoli cell biomarkers like AMH (20–22) and inhibin B (21, 23, 24), can readily inform about testicular function without the need for stimulation tests. Indeed, undetectable serum AMH is clearly more robust than testosterone post-hCG to diagnose or rule out anorchidism in boys with non-palpable gonads (25). AMH is secreted exclusively by the Sertoli cells of the testis in males, from early fetal life (26) where it is involved in male sex differentiation by inducing the regression of the Müllerian ducts, i.e., the anlagen of the uterus and Fallopian tubes. Although this process is completed in the first trimester of the fetal period, Sertoli cells continue to secrete high amounts during infancy and childhood. Testicular AMH production varies with age; therefore, the normal reference ranges of serum AMH levels change during postnatal development (27–29).

The aim of this study was to assess testicular function in prepubertal children with cryptorchidism before orchiopexy, by determining the serum concentration of AMH. Secondarily, we evaluated the serum concentrations of gonadotropins and testosterone and looked for associations between testicular function and the clinical characteristics of cryptorchidism.

## Subjects and Methods

### Study Design and Setting

We performed a retrospective, cross-sectional, analytical study at the Division of Endocrinology of the Ricardo Gutiérrez Children’s Hospital, a tertiary pediatric public hospital in Buenos Aires, Argentina.

The same pediatric endocrinologist performed a careful review of clinical charts. Clinical description of cryptorchidism as unilateral and bilateral, testicular volume measured by comparison to Prader’s orchidometer, position of the gonads, pubic hair, and genital development according to Marshall and Tanner (30), and of the presence of hernia or micropenis were extracted from the history chart. Patients’ personal history data, including gestational age and birth weight, history of hCG treatments, and orchiopexy were registered. Hormonal values were extracted from the history chart and the laboratory Cobas^®^ Infinity system (Roche).

### Patients

#### Patients With Cryptorchidism

All clinical charts of subjects admitted at the outpatient clinic of the Division of Endocrinology of the Ricardo Gutiérrez Children’s Hospital between 2000 and 2017, and recorded in our database with the diagnosis of cryptorchidism, were eligible. Cryptorchidism was defined by the absence of one or both testes in the scrotum. Inclusion criteria were the presence of cryptorchidism, normal virilization (urethral orifice at the end of the penis and complete fusion of the scrotum) and the availability of a result of serum AMH determination performed before orchiopexy at a prepubertal stage, defined by testicular volume ≤ 3 ml as compared with Prader’s orchidometer and according to Tanner stages. The following exclusion criteria applied: history chart absent or incomplete, a diagnosis of disorders of sex development or genetic syndromes known to affect testicular function, genital, or abdominal–pelvic surgeries susceptible of affecting the gonadal vessels performed before the first hormonal evaluation, radiotherapy, or chemotherapy.

#### Healthy Controls

For comparison, we used serum levels of AMH, testosterone, FSH, and LH from a sample of 179 apparently normal prepubertal boys, which have been published previously (29). This cohort fulfilled the following criteria: (i) written informed consent was given by the participant’s parents, (ii) a blood sample was being drawn for routine clinical evaluation independently of the research study, (iii) anamnesis ruled out cryptorchidism or other genital or urologic malformations, endocrine diseases, and chronic or acute general pathologies that could affect endocrine function, and (iv) a clinical examination was performed to assess Tanner pubertal stage and to determine testicular position and volume by comparison with Prader’s orchidometer.

### Outcome Measures and Definitions

The main outcome measure of the study was the serum concentration of AMH. Secondary outcome measures were serum concentrations of LH, FSH, and testosterone. Circulating levels of reproductive hormones were compared between patients with cryptorchidism and normal boys. Serum AMH and FSH were, respectively, used as a direct and an indirect biomarker of the functional mass of prepubertal Sertoli cells (21), whereas serum testosterone and LH were, respectively, used as a direct and an indirect biomarker of Leydig cells. All data were obtained at first referral, before any hormonal or surgical treatment was attempted. In a small subset of patients, a second assessment was available after orchiopexy in these cases, a longitudinal comparison (at referral vs after surgery) was made.

For the primary analysis, patients with cryptorchidism and controls were grouped by age intervals (all prepubertal). Subsequent stratification for subgroup analysis was done according to the clinical characteristics of the cryptorchidism, such as palpability and position of the testes. According to the AMH values, patients were classified as functionally anorchid when serum AMH was non-detectable, hypogonadal when serum AMH was detectable but below the normal reference level (below the 3rd percentile for age), and eugonadal when serum AMH was within the normal reference level.

Other potentially associated variables considered in this study were as follows: gestational age at birth, birth weight, penile size, presence of inguinal hernia, and hCG treatment for cryptorchidism. Gestational age was a dichotomic variable (preterm/full-term), considering preterm birth when gestational age was <37 weeks. Birth weight was analyzed as a continuous variable. Weight for gestational age was a dichotomic variable (small for gestational age/adequate for gestational age), considering small for gestational age when weight was <3rd percentile for gestational age according to local references (31). Penile size was dichotomized (micropenis/normal), and micropenis was defined by penile size < −2 SD according to the Argentine references for age (32). Presence of inguinal hernia was dichotomized (yes/no), according to the attending physician’s description in the clinical chart. Treatment with hCG followed a standardized protocol used at the Division of Endocrinology, consisting of 1,000 IU of hCG administered IM once weekly for 5 weeks, and assessment within 1 month following the last injection. Treatment was considered successful if the gonad was in the scrotum at physical examination, and unsuccessful if it was not in scrotal position.

### Study Size

The sample size was calculated for the main outcome measure, i.e., the prevalence of hypogonadism (patients with serum AMH below the normal reference range) in prepubertal patients with cryptorchidism. The estimated study size required 181 patients with bilateral cryptorchidism and 116 patients with unilateral cryptorchidism, to detect a prevalence of hypogonadism of 36 and 18%, respectively, based on previously unpublished own studies, with an accuracy of 7% and a confidence level of 95%.

### Hormone Assays

#### Anti-Müllerian Hormone

Results of serum AMH determinations were all obtained with an enzyme-linked immunoassay specific for human AMH (EIA AMH/MIS^®^, Beckman-Coulter Co., Marseilles, France), as previously validated by our group (29, 33). Intra- and inter-assay coefficients of variation were, respectively, 10.5 and 9.4%, for a serum AMH concentration of 700 pmol/L, and 11.1 and 12.8% for a serum AMH concentration of 7 pmol/L. When serum AMH levels were undetectable, the value of the limit of quantification (functional sensitivity = 2.5 pmol/L) was attributed.

#### Gonadotropins

LH and FSH were determined using electrochemiluminescent immunoassays (ECLIAs, Roche Diagnostics GmbH, Mannheim, Germany) as described (33). The limits of quantification of both LH and FSH assays were 0.10 IU/L, according to the 2nd NIBSC IS 80/552 for LH and the 2nd WHO IRP 78/549 for FSH. Intra- and inter-assay coefficients of variation were 1.1 and 1.8% for LH, respectively, for a mean LH concentration of 2.8 IU/L and 1.4 and 1.5% for a mean LH concentration of 16.9 IU/L. Intra- and inter-assay coefficients of variation were 1.0 and 4.2% for FSH, respectively, for a mean FSH concentration of 14.8 IU/L and 1.1 and 4.1% for a mean FSH concentration of 23.4 IU/L. When serum LH or FSH levels were undetectable, the value of the limit of quantification (functional sensitivity) was attributed.

#### Testosterone

Testosterone was determined in serum using an ECLIA (Roche Diagnostics GmbH, Mannheim, Germany) as described (29). Intra- and inter-assay coefficients of variation were 2.4 and 2.6%, respectively, for a mean testosterone concentration of 176 ng/dL (6.10 nmol/L) and 1.2 and 2.3% for a mean testosterone concentration of 455 ng/dL (15.78 nmol/L). When serum testosterone levels were undetectable, the value of the limit of quantification (functional sensitivity = 10 ng/dL) was attributed.

### Statistical Analyses

Because serum AMH varies with age in normal boys during infancy and childhood, values were analyzed using the standard deviation score (SDS) for age in the overall evaluation. In the age subgroup analyses, absolute serum levels and percentiles were used. Data distribution was assessed for normality using the Shapiro–Wilk test. Results are expressed as median and range. Because non-Gaussian distribution was found in most cases, non-parametric tests were used for comparisons. The Wilcoxon Signed Rank Test was used to compare median SDS with the theoretical value of 0 SDS. Mann–Whitney test was used to compare serum hormone levels between two independent groups. Kruskal–Wallis test with Dunn’s multiple comparison posttest was used when more than two groups were compared. Fisher’s exact test was used to compare categorical variables. Logistic regression was performed to analyze potential risk factors associated with decreased AMH, considered as a categorical variable (serum AMH below the 3rd percentile for age). The level of significance was set at *P* < 0.05. All statistical analyses were performed using GraphPad Prism version 7.03 for Windows (GraphPad Software, San Diego, CA, USA) and STATA 13 (StataCorp LLC, College Station, TX, USA).

## Results

### Characteristics of the Study Population

**Figure 1:**
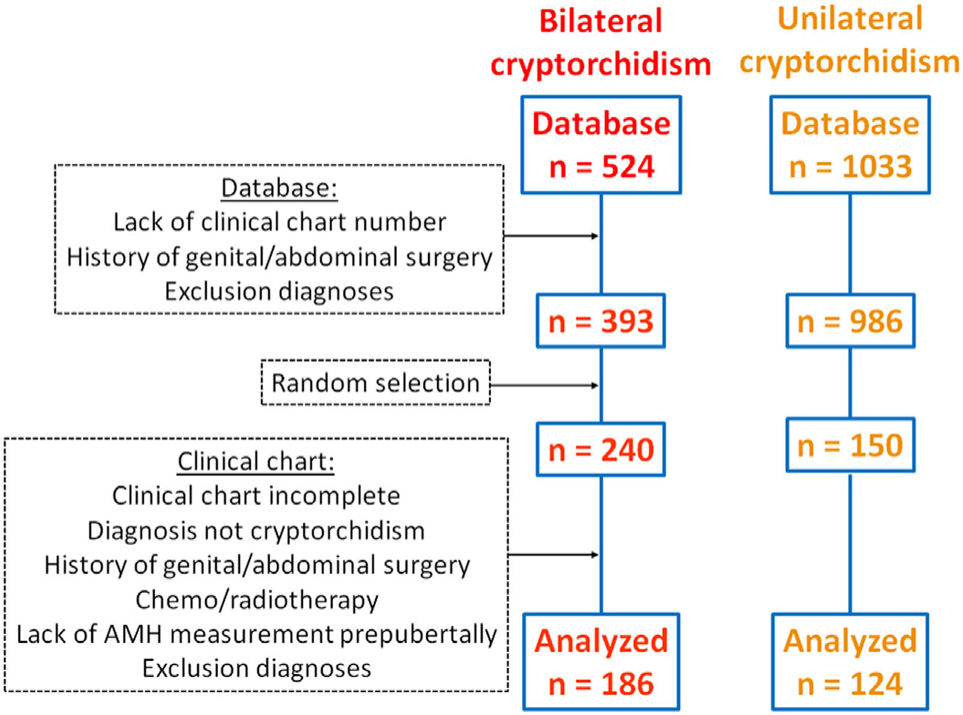
Flowchart of patient inclusion in the study.

Our database contained 1,557 patients with “cryptorchidism” as a diagnosis (524 bilateral and 1,033 unilateral cryptorchidism). Filtering the database for lack of clinical chart number, history of genital or abdominal surgery and for other exclusion diagnoses (see Subjects and Methods), a total of 178 cases were excluded, leaving 393 cases of bilateral and 986 cases of unilateral cryptorchidism as eligible for our study. Using a randomization software, we selected and reviewed 390 clinical charts (240 with bilateral and 150 with unilateral cryptorchidism). Eighty (54 with bilateral and 26 with unilateral cryptorchidism) were discarded because the history chart was incomplete, the diagnosis was not cryptorchidism (database recording error), there was a history of genital or abdominal surgery or of chemotherapy or radiotherapy reported in the chart, a result of a serum AMH measurement was not available in the prepubertal period, or other exclusion diagnoses were found in the chart. Finally, 310 patients with cryptorchidism, 186 with bilateral cryptorchidism, and 124 with unilateral (50% right cryptorchidism) were analyzed in the study (Figure 1).

Median age at first hormonal evaluation was approximately 3 years, with a wide range (Table 1). The proportion of patients born preterm, with micropenis or hernia, or having received hCG treatment for cryptorchidism varied according to the age groups. Only two preterm patients were born at <28 weeks.

**Table 1:** Clinical characteristics of patients with unilateral or bilateral cryptorchidism included in this study.

	All ages		1–5.9 months		6 months–1.9 years		2–8.9 years		≥9 years	
	Unilateral	Bilateral	Unilateral	Bilateral	Unilateral	Bilateral	Unilateral	Bilateral	Unilateral	Bilateral
*n*	124	186	12	9	28	54	68	100	16	23
Age, median (range)	3.06 (0.03–11.10)	3.33 (0.06–13.62)	0.39 (0.03–0.47)	0.31 (0.06–0.49)	1.26 (0.55–1.97)	1.21 (0.54–1.94)	4.01 (2.00–8.98)	4.65 (2.02–8.67)	10.18 (9.06–11.12)	10.47 (9.04–13.62)
Preterm, *n* (%)	11 (8.9)	25 (13.4)	1 (8.3)	0	2 (7.1)	8 (14.8)	5 (7.4)	13 (13.0)	3 (18.8)	4 (17.4)
Micropenis, *n* (%)	3 (2.4)	14 (7.5)	1 (8.3)	2 (22.2)	0	5 (9.3)	1 (1.5)	7 (7.0)	1 (6.3)	0
Hernia, *n* (%)	32 (25.8)	31 (16.7)	3 (2,500)	2 (22.2)	9 (32.1)	8 (14.8)	18 (26.5)	17 (17.0)	2 (12.5)	3 (13.0)
hCG treatment, *n* (%)	43 (34.7)	89 (47.9)	2 (16.7)	2 (22.2)	7 (25.0)	19 (35.2)	27 (39.7)	55 (55.0)	7 (43.8)	13 (56.5)

### Anti-Müllerian Hormone

**Figure 2:**
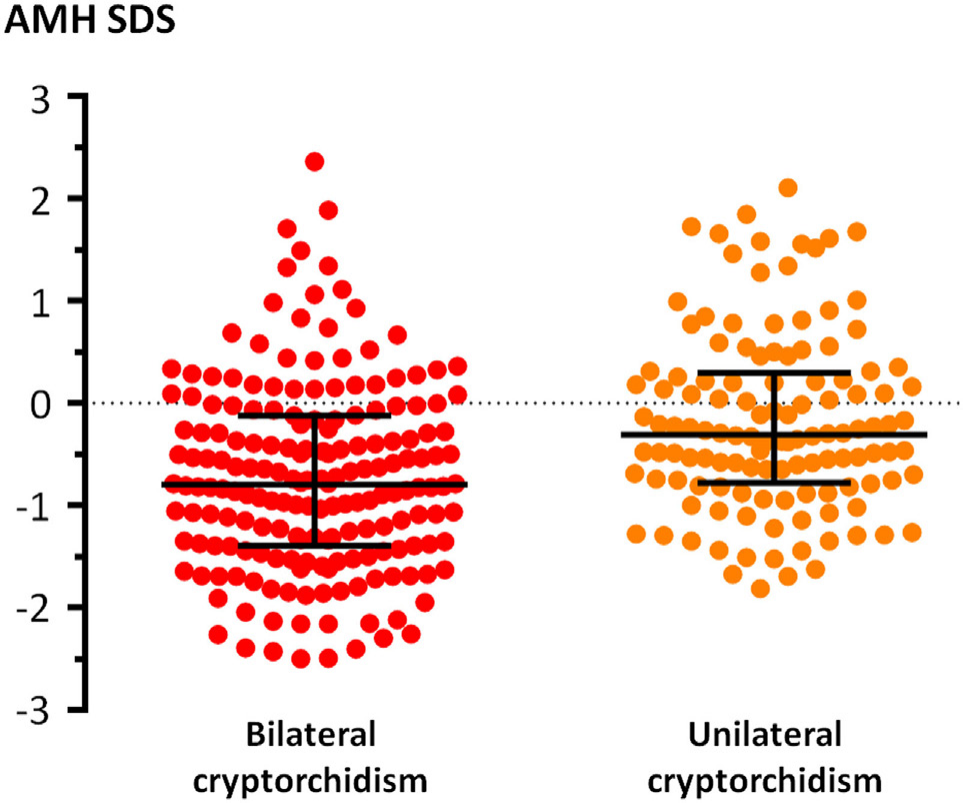
Serum levels of anti-Müllerian hormone (AMH), expressed as standard deviation score (SDS) for age, in patients with unilateral or bilateral cryptorchidism. Bars indicate medians and interquartile ranges.

Median AMH SDS was below 0 in both the bilaterally (Wilcoxon signed rank test, *P* < 0.0001) and the unilaterally (*P* = 0.0052) cryptorchid groups (Figure 2), indicating that testicular function is overall decreased in patients with cryptorchidism. Testicular function was more affected in the bilaterally than in the unilaterally cryptorchid group (Mann–Whitney test, *P* < 0.0001). In the bilaterally cryptorchid boys, serum AMH was below 0 SDS in 80.1% of the cases; furthermore, it was below −1 SDS in 39.8% of the cases. In the unilateral cryptorchidism group, the impairment was milder, with only 62.9% of the patients with serum AMH below 0 SDS.

**Figure 3:**
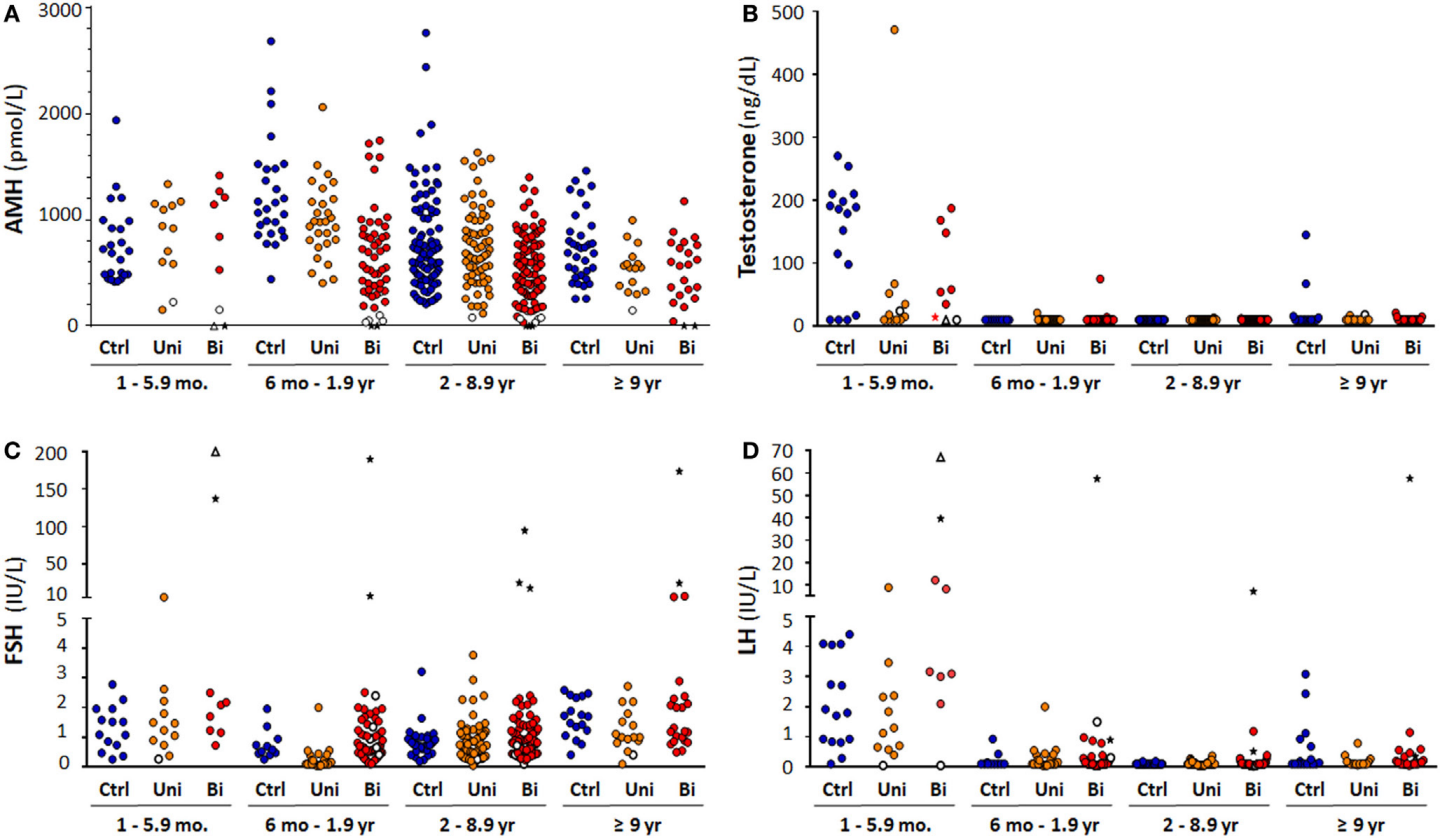
Hormone serum levels in cryptorchid boys: **(A)** anti-Müllerian hormone (AMH), **(B)** testosterone, **(C)** FSH, and **(D)** LH. Empty circles indicate cryptorchid boys with micropenis, stars indicate anorchid boys, and triangles indicate anorchid boys with micropenis. Abbreviations: Ctrl, normal controls; Uni, unilateral cryptorchidism; BI, bilateral cryptorchidism.

To assess testicular function specifically in patients with unilateral or bilateral cryptorchidism according to age, we analyzed absolute serum AMH levels and the percentile distribution. In patients with bilateral cryptorchidism, serum AMH was undetectable—thus indicative of anorchidism—in nine cases (Figure 3A). This represents 4.8% of all patients with bilateral cryptorchidism and 26.5% of those with non-palpable gonads. Patients with anorchidism were excluded from all the subsequent comparisons of testicular function between groups. Serum AMH concentration of boys with bilateral cryptorchidism was significantly lower than in the control and the unilaterally cryptorchid groups between 6 months and 1.9 years and between 2 and 8.9 years of age (Table 2).

**Table 2:** Serum hormone levels in normal controls and in patients with unilateral or bilateral cryptorchidism.

	1–5.9 months			6 months–1.9 years			2–8.9 years			≥9 years		
	Controls	Unilateral	Bilateral	Controls	Unilateral	Bilateral	Controls	Unilateral	Bilateral	Controls	Unilateral	Bilateral
**AMH^a^**												
*n*	24	12	7	26	28	52	95	68	97	34	16	21
Median (pmol/L)	697	931	1,145	1,132	981	563	684	682	525	713	551	580
Range (pmol/L)	418–1,939	153–1,337	153–1,417	441–2,682	405–2,061	34–1,747	207–2,761	80–1,635	31–1,400	256–1,462	147–966	43–1,175
<3rd percentile, *n* (%)		2 (16.7)	1 (14.3)		2 (7.1)	19 (36.5)		5 (7.2)	18 (18.6)		1 (6.3)	2 (9.5)
Kruskal–Wallis + Dunn^b^	a	a	a	a	a	b	a	a	b	a	a	a

**FSH**												
*n*	14	12	7	11	28	52	33	66	90	17	16	21
Median (IU/L)	1.30	1.36	1.70	0.59	0.10	0.71	0.75	0.84	0.83	1.70	1.0	1.20
Range (IU/L)	0.26–2.78	0.27–6.16	0.73–2.5	0.26–1.96	0.05–2.00	0.10–2.51	0.20–3.21	0.05–3.77	0.10–2.40	0.41–2.59	0.10–2.72	0.50–7.33
>97th percentile, *n* (%)		1 (8.3)	0		1 (3.6)	3 (5.8)		1 (1.5)	0		1 (6.3)	4 (19.1)
Kruskal–Wallis + Dunn^b^	a	a	a	a	a	a	a	a	a	a	a	a

**LH**												
*n*	15	12	7	11	28	52	34	67	90	17	16	21
Median (IU/L)	1.8	1.215	3.1	0.1	0.1	0.1	0.1	0.1	0.1	0.1	0.1	0.1
Range (IU/L)	0.10–4.41	0.10–8.90	0.10–12.20	0.10–0.93	0.10–2.00	0.10–1.50	0.10–0.19	0.10–0.37	0.10–1.19	0.10–3.09	0.10–0.79	0.10–1.15
>97th percentile, *n* (%)		1 (8.3)	2 (22.2)		1 (3.6)	3 (5.8)		7 (10.6)	6 (6.7)		0	0
Kruskal–Wallis + Dunn^b^	a	a	a	a	a	a	a	a	a	a	a	a

*^a^To obtain serum anti-Müllerian hormone (AMH) in ng/mL, divide by 7.14*.

*^b^Kruskal–Wallis test followed by Dunn’s multiple comparison test (Controls vs Unilateral vs Bilateral). In each age subgroup, a different letter indicates that there is a significant difference when comparing Controls vs Unilateral vs Bilateral (*P* < 0.05)*.

The prevalence of serum AMH below the normal range (<3rd percentile) indicated an increased proportion of patients with hypogonadism in all age groups, even in those groups with a normal median serum AMH (Table 2). The prevalence of AMH below the normal range was greater in patients with bilateral cryptorchidism than in boys with unilateral cryptorchidism between 6 months and 1.9 years (Fisher’s exact test, *P* = 0.006) and in boys between 2 and 8.9 years (Fisher’s exact test, *P* = 0.043). In fact, AMH was below the normal range in 16.6% of the whole group of patients with cryptorchidism and present gonads, 22.6% of boys with bilateral cryptorchidism and 8.1% of boys with unilateral cryptorchidism. These results showed only minor changes when patients with central hypogonadism were excluded (Table S1 in Supplementary Material).

Testicular function was not worse in patients with bilaterally non-palpable gonads, as compared with patients with bilateral cryptorchidism and at least one palpable gonad (Fisher’s exact test, *P* = 0.45). Median serum AMH was not significantly different in any of the age groups (Table 3).

**Table 3:** Serum anti-Müllerian hormone (AMH) in bilaterally cryptorchid boys with non-palpable gonads and boys with at least one palpable gonad.

	1–5.9 months		6 months–1.9 years		2–8.9 years		≥9 years	
	Non-palpable gonads	At least one palpable gonad	Non-palpable gonads	At least one palpable gonad	Non-palpable gonads	At least one palpable gonad	Non-palpable gonads	At least one palpable gonad
*n*	1	6	9	43	12	85	3	18
Median (pmol/L)	1,269	993	530	574	498	535	260	593
Range (pmol/L)	N.A.	153–1,417	277–1,003	34–1,747	35–938	31–1,400	43–835	178–1,175
Mann–Whitney test	N.A.		a	a	a	a	a	a

*N.A., not applicable*.

*To obtain serum AMH in ng/mL, divide by 7.14*.

*Mann–Whitney test (“Non-palpable gonads” vs “At least one palpable gonad”): same letter indicates lack of significant difference (*P* > 0.05)*.

**Figure 4:**
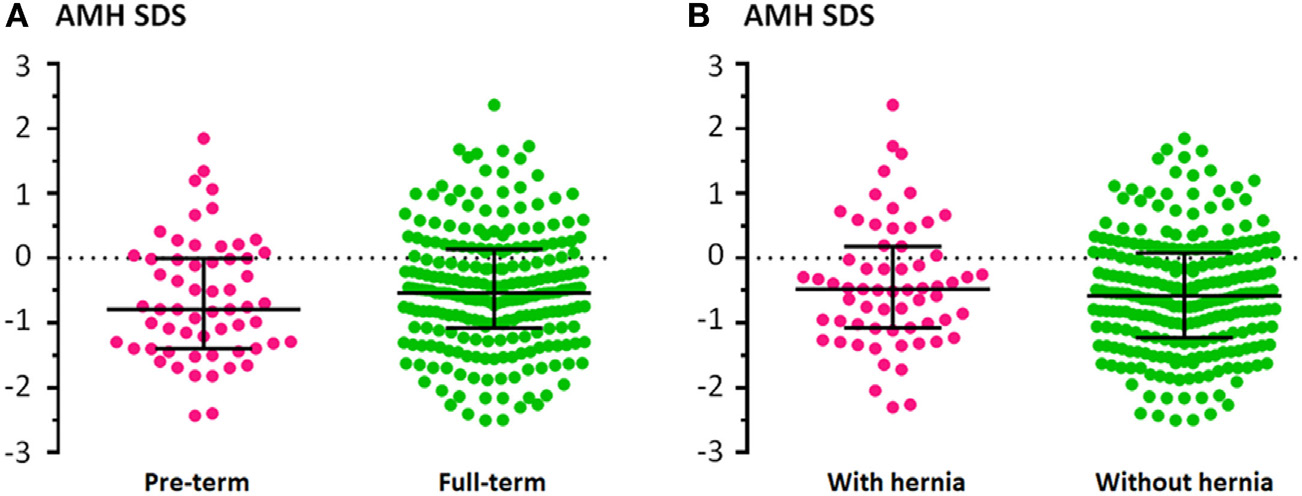
Serum levels of anti-Müllerian hormone (AMH), expressed as standard deviation score (SDS) for age, in preterm or full-term patients with unilateral or bilateral cryptorchidism **(A)** and in unilaterally and bilaterally cryptorchid patients with or without hernia **(B)**. Bars indicate medians and interquartile ranges.

To identify risk factors for hypogonadism (AMH levels < 3rd percentile) in cryptorchid boys, we performed a logistic regression (Table 4). The factors that were significantly associated with AMH levels < 3rd percentile were bilateral cryptorchidism, as compared with unilateral cryptorchidism, and the presence of micropenis. Sixteen out of 17 boys (94.2%) with micropenis had AMH levels below the normal range (Figure 3A; Table 5). One patient was anorchid (undetectable AMH) and five of them were diagnosed with central hypogonadism (data obtained from clinical charts reporting testosterone treatment at age ≥14 years). There were no significant differences in the prevalence of hypogonadism (AMH < 3rd percentile) between preterm and full-term patients, either in the total group or in patients with unilateral or bilateral cryptorchidism (Table 6). Likewise, serum AMH did not differ significantly between preterm and full-term cryptorchid patients (Mann–Whitney test, *P* = 0.275; Figure 4A). We hypothesized that, in patients with hernia, testicular maldescent would be the result of an anatomical hindrance rather than a testicular dysfunction. However, the prevalence of hypogonadism was not significantly lower than that observed in patients with cryptorchid gonads not associated with an inguinal hernia (Table 6). Also, no difference was found in serum AMH between cryptorchid patients with or without inguinal hernia (Mann–Whitney test, *P* = 0.288; Figure 4B).

**Table 4:** Logistic regression performed to identify potential risk factors for hypogonadism (AMH levels < 3rd percentile) in boys with cryptorchidism (unilateral and bilateral considered together).

	Odds ratio	95% CI	*P*
Bilateral cryptorchidism	3.63	1.52–8.66	0.004
Micropenis	91.70	10.96–767.05	<0.001
Hernia	1.41	0.60–3.3327	0.430
Preterm	0.81	0.27–2.41	0.705
Birth weight	0.99	0.99–1.00	0.389
SGA	0.54	0.15–1.91	0.339
Age at evaluation	1.02	0.91–1.14	0.748

*CI, confidence interval; AMH, anti-Müllerian hormone; SGA, small for gestational age*.

**Table 5:** Serum hormone levels in cryptorchid boys with micropenis.

	1–5.9 months	6 months–1.9 years	2–8.9 years	≥9 years
**AMH^a^**				
*n*	3	5	8	1
Median (pmol/L)	153	53	79	147
Range (pmol/L)	N.D.–226	34–321	31–525	N.A.
<3rd percentile, *n* (%)	3 (100.0)	5 (100.0)	7 (87.5)	1 (100.0)
Anorchia	1	0	0	0
Bilateral/unilateral	2/1	5/0	8/0	0/1

**FSH**				
*n*	3	5	8	1
Median (IU/L)	0.73	0.94	0.45	0.41
Range (IU/L)	0.27–200	0.42–2.4	0.10–0.74	N.A.
>97th percentile, *n* (%)	1 (33.3)	1 (20.0)	0	0

**LH**				
*n*	3	5	8	1
Median (IU/L)	0.1	0.1	0.1	0.1
Range (IU/L)	0.10–67.09	0.1–1.5	0.10–0.10	N.A.
>97th percentile, *n* (%)	1 (33.3)	1 (20.0)	0	0

*N.A., not applicable*.

*^a^To obtain serum AMH in ng/mL, divide by 7.14*.

**Table 6:** Prevalence of patients with hypogonadism (anti-Müllerian hormone levels < 3rd percentile) according to prematurity and the presence of hernia in cryptorchid boys.

	Preterm	Term	*P*^a^	Hernia	No hernia	*P*^a^
All, *n* (%)	10 (27.8)	39 (15.4)	0.093	11 (17.5)	48 (19.4)	0.858
Unilateral cryptorchidism, *n* (%)	2 (18.2)	8 (7.3)	0.303	2 (6.3)	8 (8.7)	1.000
Bilateral cryptorchidism, *n* (%)	8 (32.0)	31 (22.0)	0.229	9 (28.0)	40 (26.0)	0.826

^a^Fisher’s exact test, “Preterm” vs “Term” or “Hernia” vs “No hernia.”

**Figure 5:**
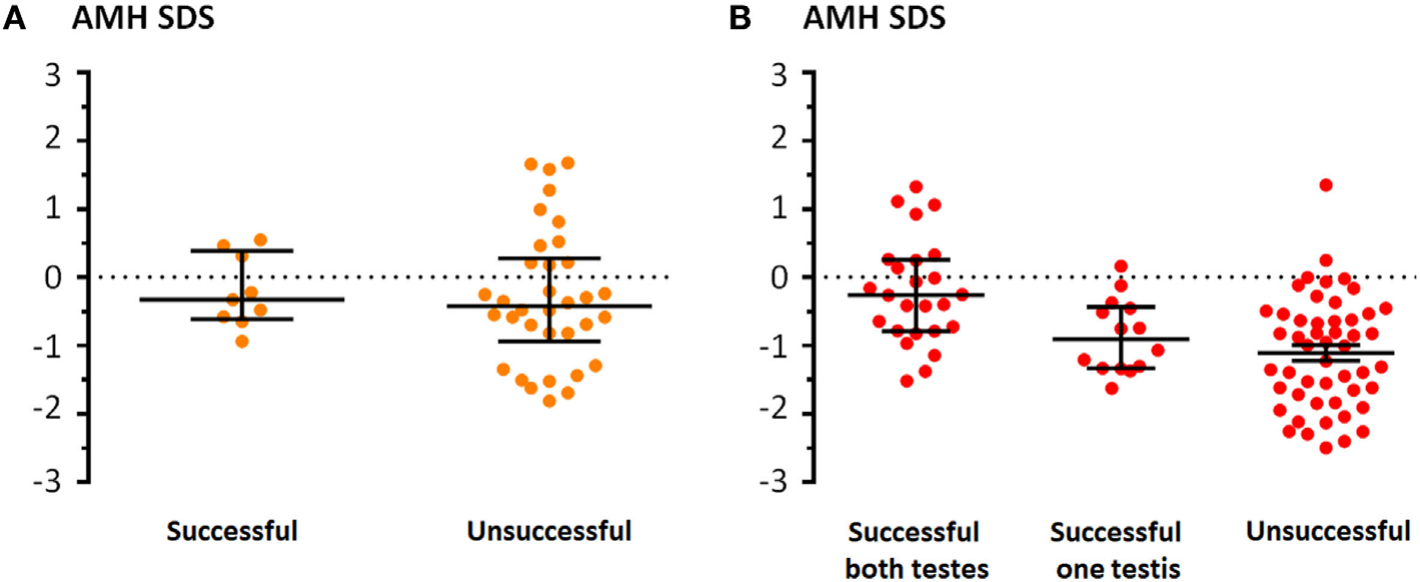
Serum levels of anti-Müllerian hormone (AMH), expressed as standard deviation score (SDS) for age, in patients who received hCG treatment for cryptorchidism. **(A)** Unilateral cryptorchidism. **(B)** Bilateral cryptorchidism. Successful indicates that the testis was in scrotal position at physical examination in the visit following hCG treatment.

Treatment with hCG for cryptorchidism was performed in 132 of 310 (42.6%) patients according to local standard procedures. Treatment was performed in 43 of the 124 (34.7%) patients with unilateral cryptorchidism, with a success rate of 20.9%. AMH levels did not differ significantly between patients in whom hCG treatment was successful or unsuccessful (Mann–Whitney test, *P* = 0.581; Figure 5A). In the group of bilaterally cryptorchid boys, 89 of 186 (47.9%) received hCG with a success rate of 28.1% for the descent of both testes and 15.7% for one testis. AMH levels of bilaterally cryptorchid boys who showed a successful response of both testes to hCG were higher than those of boys with no response (Kruskal–Wallis test followed by Dunn’s Multiple Comparison Test, *P* = 0.0001; Figure 5B).

**Figure 6:**
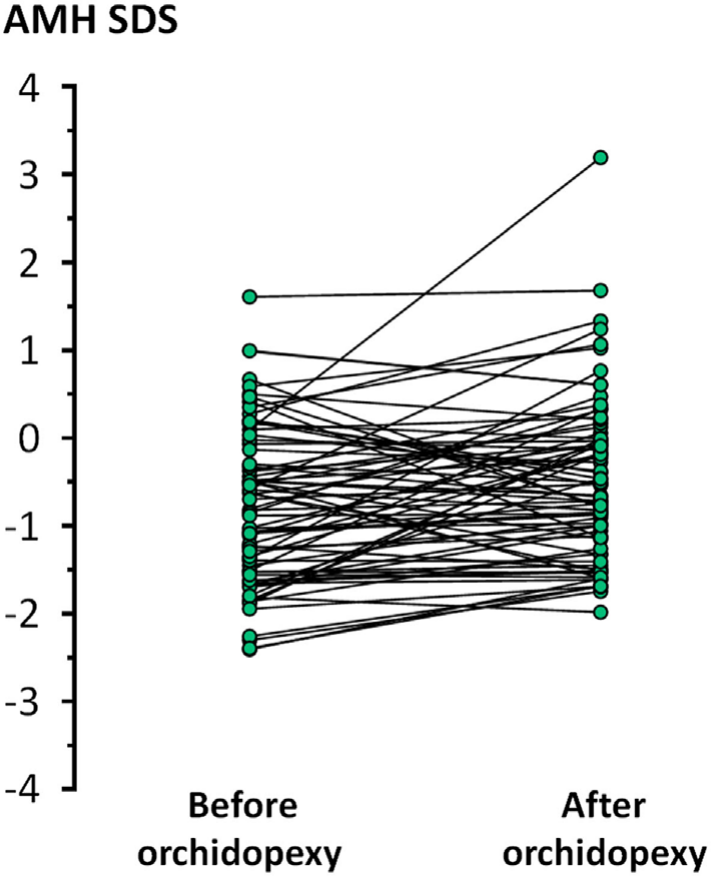
Serum levels of anti-Müllerian hormone (AMH), expressed as standard deviation score (SDS) for age, at referral and at least 1 month after orchidopexy in patients with cryptorchidism (unilateral or bilateral).

Orchiopexy was performed in 151 patients. Serum AMH levels were available in 76 patients at referral, i.e., before any treatment was attempted, and at least 1 month after surgery. A statistically significant increase was observed in AMH levels after orchiopexy (Paired *T* test, *P* = 0.0030, Figure 6).

### Testosterone

In patients aged 1–5.9 months, lower testosterone levels were observed in the unilaterally cryptorchid group as compared with controls (Kruskal–Wallis test followed by Dunn’s Multiple Comparison Test, *P* = 0.029; Figure 3B). During the rest of childhood, testosterone levels are usually very low or undetectable in normal boys. Accordingly, serum testosterone was below the limit of detection of the assay (10 ng/dL) in 265 of 276 cryptorchid patients aged >6 months; in the remaining 11 patients, serum testosterone ranged between 11 and 75 ng/dL. Therefore, no statistical comparisons were made between groups.

### Gonadotropins

Serum gonadotropin levels were within the normal range in the vast majority of patients with unilateral or bilateral cryptorchidism independent of age (Figures 3C,D). Only 4 of 124 (3.2%) boys with unilateral cryptorchidism had elevated FSH (>97th percentile for age), between 2.7 and 6.2 IU/L. In boys with bilateral cryptorchidism, FSH was elevated in 15 of 186 (8.1%) cases. Eight of them proved to be anorchid, with FSH levels ranging from 7 to 200 IU/L. In the remaining seven cases, FSH was mildly elevated, between 2 and 7.3 IU/L.

Serum LH was elevated (>97th percentile for age), between 0.2 and 8.9 IU/L, in 9 of 124 (7.3%) boys with unilateral cryptorchidism, and in 17 of 186 (9.1%) boys with bilateral cryptorchidism (Figure 3). Seven of the latter were anorchid, with LH levels ranging from 0.25 to 67.1 IU/L. In the remaining 10 cases, LH was mildly elevated, between 0.2 and 12.2 IU/L.

When the subgroup of patients with abnormally low AMH (<3rd percentile for age) was analyzed, 13 of 59 (22.0%) had elevated FSH for age, 8 of whom were anorchid. In the 51 cryptorchid patients with bilaterally present gonads, elevated FSH was observed in only 5 cases (9.8%). Serum LH was elevated in 9 of the 59 patients (15.3%), and in only 2 (3.9%) of the boys with present testes.

## Discussion

Controversial results on whether testicular AMH production is decreased in prepubertal boys with cryptorchidism have been reported in a few studies with small number of patients of a large range of ages and including cases of both unilateral and bilateral cryptorchidism (25, 34–38) (Table 7). Our study including 310 prepubertal patients randomly selected from a cohort of more than 1,500 boys with cryptorchidism shows that, as a group, cryptorchid patients have lower AMH production than normal boys. Even though most of cryptorchid patients have serum AMH levels within the reference range, there is a considerable prevalence of cases with abnormally decreased serum AMH, indicating an affected testicular function during childhood. The prevalence of testicular dysfunction did not increase with age. As could be expected, low serum AMH concentration was found more often in patients with bilateral than unilateral cryptorchidism.

**Table 7:** Comparison between this work and previously published articles reporting on AMH serum or expression levels in boys with cryptorchidism.

Reference	Sample size	Population	Age	Unilateral/bilateral cryptorchidism	AMH serum or testicular expression levels
Present work	310	Argentine	0.03–13.6 years (prepubertal)	124 unilateral and 186 bilateral	Decreased in 22.6% of boys with bilateral and 8.1% of boys with unilateral cryptorchidism
(39)	156	USA	1 day–20 years	16 unilateral and 140 bilateral	Undetectable AMH in 30%, low in 21%, and normal in 49%
(40)	105	Polish	1–4 years	Unilateral	Mean AMH not statistically different from controls Gene polymorphisms in cryptorchid boys, no association with AMH levels
(41)	104	Australian	0–18 years	76 unilateral and 28 bilateral	Lower mean AMH in bilateral cryptorchid as compared with unilateral and controls
(38)	94	Danish	0.5–13.1 years (prepubertal)	53 unilateral and 41 bilateral	Decreased in 5% (no distinction made between unilateral and bilateral)
(25)	65	USA	2 days–11 years	Bilateral non-palpable	Undetectable AMH in anorchid, low AMH in 14 cryptorchid with histological damage, normal AMH in 34 with histologically normal testes
(42)	50	Polish	1–4 years	Unilateral	Mean AMH not statistically different before and after orchiopexy
(37)	50	Polish	1–4 years	Unilateral	Lower mean AMH in cryptorchid as compared with controls
(36)	43	Various	85 ± 31 days	N.S.	Mean AMH not statistically different from controls
(43)	31	Brazilian	0.75–9 years	24 unilateral and 7 bilateral	Mean AMH within normal range
(44)	27	French	14–32 months	17 unilateral and 10 bilateral	Lower mean AMH in bilateral but not unilateral cryptorchid as compared with controls
(35)	20	Turkish	12 months	20 unilateral	Lower mean AMH in cryptorchid as compared with controls
(45)	18	N.S.	0.6–6.1 years	N.S.	No differential AMH mRNA levels reported between patients with high risk and those with low risk for azoospermia
(34)	15	French	1 day–10 years	Unilateral and bilateral	Decreased in 75% (no distinction made between unilateral and bilateral)
(46)	15	N.S.	7–55 months	7 unilateral and 8 bilateral	No changes reported in AMH mRNA levels in testicular biopsy

*N.S., not specified; AMH, anti-Müllerian hormone*.

With the aim of evaluating the pituitary–testicular axis in patients with cryptorchidism, serum levels of another Sertoli cell marker—inhibin B—and of Leydig cell markers—testosterone and INSL3—have also been assessed in different studies. INSL3, but not the other markers, has consistently been found low in cord blood from cryptorchid newborns (47, 48). Basal testosterone and INSL3 are no longer informative in childhood after postnatal activation wanes at 3–6 months of age, since their circulating levels are very low or undetectable. Like AMH, inhibin B also shows controversial results in boys with cryptorchidism. However, most studies indicate that there is an increased prevalence of patients with low basal inhibin B or inhibin B/FSH ratio in older infants and children with cryptorchidism (38, 44, 49–51). Altogether, these results and our present data using basal AMH as a Sertoli cell marker suggest that there is an increased risk of seminiferous tubule dysfunction already in childhood even though the gonadotropin axis is relatively quiescent. Furthermore, lower levels of inhibin B have been shown to correlate with decreased number of spermatogonia in infancy (50, 52), a predictor of infertility in males with a history cryptorchidism (53–56).

In our large cohort of cryptorchid boys, the median SDS for serum AMH was below 0 both in the unilaterally and bilaterally cryptorchid groups, which indicates that the testicular Sertoli cell compartment is overall affected. Serum AMH levels found in patients with bilateral cryptorchidism were clustered in the lowest ranges: indeed, 39.8% of the values were below 1 SDS, as compared with 15.9% expected according to a Gaussian distribution. Furthermore, more than one-fifth of patients with bilateral cryptorchidism had overtly abnormal serum AMH, i.e., levels below the 3rd percentile for age, thus validating the prevalence of childhood hypogonadism previously reported in a smaller series of cryptorchid boys (38). Other studies report mean serum AMH levels but do not discriminate the percentage of patients with abnormally low AMH production (34, 35, 37, 44). When analyzed by age groups, we found a prevalence of hypogonadism—as indicated by AMH < 3rd percentile—in 36.5% of the patients between 6 months and 2 years, an age at which most cryptorchid patients are referred to the pediatric endocrinologist. In our series, recruited between 2000 and 2017, we have noticed a high frequency of relatively late referral, with a predominance after 2 years of age, which raises the concern of a potential progressive testicular damage until the time of treatment (57–59). However, the proportion of bilaterally cryptorchid patients with impaired Sertoli cell function was not higher in the 2- to 9-year-old group than in the younger group. Furthermore, no influence of age at first hormonal evaluation (always performed before treatment in our series) was found on the prevalence of impaired Sertoli cell function as determined by serum AMH < 3rd percentile, in agreement with other observational studies indicating no progressive damage associated with delayed orchiopexy (57, 60–62). The latter studies clearly identified that there are at least two conditions in patients with cryptorchidism: in one, testes are already affected at early infancy, showing absence of spermatogonia type Ad, and in the other, testes have Ad spermatogonia. After long-term follow-up until adulthood, patients who had Ad spermatogonia at biopsy during orchiopexy were fertile with normal spermiograms in contrast to those with absence of Ad spermatogonia, who showed abnormal spermiograms, regardless of the age at orchiopexy (57). Nonetheless, it should be emphasized that our study was not designed to assess the effect of early vs late treatment on testicular function, and definitive results of long-term prospective clinical trials will certainly shed light on this concern (58, 63). Another limitation of our study is linked with the relatively small number of patients of the two extreme age groups, which may result in low power to detect decreased AMH or increased prevalence of Sertoli cell dysfunction with enough statistical significance.

In the unilateral cryptorchidism group, the impairment was milder, with only 62.9% of the patients with serum AMH below 0 SDS and less than 10% of patients with AMH below the 3rd percentile. These results confirm results of a recent study comparing serum AMH between unilaterally cryptorchid patients and normal boys (40), and are in line with the observation in a large scale, long-term follow-up study, that unsuccessful paternity was 10.3% in patients with a history of unilateral cryptorchidism and 6.8% in controls (64).

With the aim of identifying risk factors associated with Sertoli cell dysfunction in patients with cryptorchidism, in addition to the previously discussed bilaterality, logistic regression analysis detected micropenis with a very high odds ratio. A limitation of our study, related to its retrospective design, is that certain recently detected genetic, maternal, and environmental risk factors (65) were not screened by the attending clinician. The occurrence of micropenis at birth is indicative of insufficient androgen exposure during the second half of fetal life (66), which can be due to fetal testicular regression syndrome resulting in congenital anorchidism. This was the case in one patient with non-palpable gonads of our cohort, in whom the finding of undetectable AMH in serum lead to early diagnosis of anorchidism. More frequently, congenital micropenis is associated with central (hypogonadotropic) hypogonadism. In these cases, the low levels of AMH are the consequence of insufficient FSH in intrauterine life resulting in decreased number of Sertoli cells and impaired AMH gene expression in each Sertoli cell (67–69).

Excluding anorchid patients, the majority of whom showed very high gonadotropin levels, only a very low proportion of prepubertal cryptorchid boys had a mild elevation of serum gonadotropins, even in the case of patients with manifest primary hypogonadism as revealed by abnormally low serum AMH levels, thus emphasizing that primary hypogonadism is rarely hypergonadotropic in prepubertal patients (33, 70, 71).

The pathogenesis of cryptorchidism may involve disorders of the hypothalamic–pituitary–gonadal axis or anatomical defects with no primary endocrine deficiency. In central or primary hypogonadism, low testosterone and/or INSL3 are responsible for the testicular maldescent. In anatomical defects, like an inguinal hernia, the obstruction of the inguinal canal may preclude the normal gonad from completing its descent to the scrotum. Therefore, we hypothesized that AMH would not be affected in cryptorchid patients with inguinal hernia. However, when comparing between patients with and patients without inguinal hernia, we did not find a significant difference in serum AMH levels, or in the prevalence of patients with evident Sertoli cell dysfunction (AMH < 3rd percentile). A possible explanation is that the abnormal testicular position *per se* would affect the Sertoli cell population, independent of the pathogenic mechanism underlying cryptorchidism.

The overtly low AMH levels detected in a subset of patients with cryptorchidism could be associated with one or more possibilities. One is that fetal testicular development is primarily defective, and low AMH behaves as a biomarker. Evidence for a primary testicular defect arises from large association studies showing that reproductive conditions observed at birth, like cryptorchidism and hypospadias, and in adults, e.g., low sperm counts and testicular cancer, are increasing in incidence concomitantly, and are signs of the so-called testicular dysgenesis syndrome (72). Furthermore, the risk of testicular cancer in patients with a history of unilateral cryptorchidism is increased in both testes, indicating that in addition to the ectopic position of the testis, there are other preexisting factors involved in the mechanism underlying the association between cryptorchidism and testicular cancer (73). Another possibility is that the abnormal position of the testes impairs Sertoli cell development. Normally, Sertoli cells proliferate during the postnatal activation of the gonadal axis (74) resulting in a mild but significant increase in testicular volume (75, 76) and serum AMH (27–29). Conversely, in cryptorchid boys the increase in Sertoli cell number (58, 77) and testicular volume (58) is impaired. The increase in serum AMH levels after orchiopexy observed in our study suggests that testicular damage might be at least partially reversible. Finally, decreased AMH has been postulated as one etiologic factor for cryptorchidism.

In fact, in addition to testosterone and INSL3, AMH has been proposed to be involved in testicular descent on the basis of observations made in patients with persistent Müllerian duct syndrome (PMDS) due to mutations in the genes coding for either AMH or its specific receptor AMHR2, although an experimental proof-of-concept is still lacking (13). Because the testes remain in ovarian position in approximately 40% of PMDS boys (78), AMH was initially thought to be involved in the first (transabdominal) phase of testicular descent as a candidate to stimulate the swelling reaction in the gubernaculum (79), until INSL3 was discovered as the factor controlling gubernaculum shortening (80). Furthermore, normal testicular descent was observed in mice with an experimental knockout of the *Amh* (81) or the *Amhr2* (82) gene and in male pups of a female rabbit with high levels of blocking anti-AMH antibodies (83). Altogether, these observations favored the hypothesis that cryptorchidism in PMDS patients is related to the anatomical attachment of the testes to the broad ligaments of the persistent uterus (78). However, there are anatomical differences that exist between humans and mammalian species used as experimental models (13), and two anatomical forms responsible for about 60% of PMDS cases, presenting either with unilateral cryptorchidism where either one testis in inguinal position along with its attached tube and uterus (known as *hernia uteri inguinalis*) or with both testes and Müllerian derivatives herniated on the same side (known as transverse testicular ectopia), show an abnormally long gubernaculum. These observations, consistent with a role for AMH gubernacular shrinkage, have led the hypothesis that AMH may increase INSL3 action on the gubernaculum by stimulating the shortening of the gubernacular cord (13). This hypothesis needs experimental evidence. Our observational study design was not conceived to address the potential implication of AMH in testicular descent in the largely most frequent cases of cryptorchidism, i.e., non-PMDS patients.

The choice between surgical and hormonal treatment for cryptorchidism has raised controversies in the last decade (84, 85), that go beyond the scope of this work. A meta-analysis demonstrated that the lower the original position of the testis, the better the effect of the hormonal treatment, thus suggesting that LHRH or hCG can be tried in the treatment of inguinal or high scrotal position (86). In our series of patients with bilateral cryptorchidism, serum AMH concentration was higher in those with a bilaterally successful testicular descent in response to a 5-week hCG treatment protocol than in those with no response. These results are in line with the concordance observed between serum AMH and testosterone concentrations during hCG stimulation in boys undergoing gonadal function assessment (87), and suggest that basal AMH determination may serve as predictive marker of response to hCG treatment, in addition to testicular position.

The large sample size of cryptorchid patients included in this study and the randomized method used to select cases from a database of more than 1,500 potentially eligible patients is one major strength of this work. We acknowledge the existence of the possibility of a selection bias related to the fact that our study was performed in the endocrinology service of a tertiary hospital. Cryptorchidism is a condition traditionally treated by surgeons. However, the rate of referral of cryptorchid patients to the endocrine unit is high in our hospital, due to the multidisciplinary approach that has been implemented in the last two decades and deems endocrine assessment as essential in the management of patients with cryptorchidism. The relatively low number of patients in the 1–5.9 and >9 years precluded certain analyses with the desired power, yet reflect that a low number of patients are referred in the first months of life, awaiting a potential spontaneous descent (1, 4, 5, 88) but that most are referred before pubertal onset, as expected.

Our study included patients over a period of 18 years (years 2000 through 2017) but had a cross-sectional design, which precluded us from performing longitudinal analyses. Another concern that may arise from such a long study whose main outcome measure derives essentially from AMH determination is related to changes in the hormone assay methodology. Because we were involved in the development of the commercial AMH/MIS Beckman-Immunotech assay [usually referred to as IOT (89, 90)], we have been able to use the same assay all through this study. The applicability of our results is assured, even though the IOT assay is no longer available, because results of serum AMH in the male range are comparable with those obtained with the newly developed AMH assays (91).

In conclusion, the population of prepubertal boys with cryptorchidism have lower AMH production than normal boys, especially those with bilaterally undescended gonads. This could be the result of a decreased number of Sertoli cells in the cryptorchid gonads and/or to an impaired AMH secretion by each Sertoli cell. Although cryptorchid boys may have serum AMH within the normal range, our results in a large cohort, together with those previously reported in smaller studies, indicate that there is a considerable prevalence of testicular dysfunction during childhood in this frequent condition. The proportions of unilaterally or bilaterally cryptorchid patients with decreased AMH in our study are consistent with the risk of azoospermia in adulthood (45). However, to know whether Sertoli cell dysfunction early in life underlies germ cell failure in adulthood will need longitudinal follow-up for many years.

## Ethics Statement

The study protocol was approved by the Institutional Review Board (Comité de Docencia e Investigación) and Ethics Committee (Comité de Ética en Investigación) of the Buenos Aires Children’s Hospital (Hospital de Niños Ricardo Gutiérrez de Buenos Aires). Because the study of patients with cryptorchidism was based on a retrospective clinical chart review with descriptive purposes and no anticipated effect on prognosis or therapeutic management of the patients whose charts were included, the need for a written informed consent was waived. For the control group, written informed consent was given by the participant’s parents, and assent was given by the participants over 7 years of age.

## Author Contributions

RG and RR conceived the study design and drafted the manuscript; all the authors collected clinical and laboratory data and approved the final version; RG analyzed the data.

## Conflict of Interest Statement

RR has received royalties derived from an agreement between INSERM (France) and Beckman-Coulter-Immunotech for the development of the AMH ELISA. RR, RG, and PB have received honoraria from CONICET (Argentina) for technology services using the AMH ELISA. SG has no conflicts of interest to disclose.
